# DNA Damage and Repair in Schizophrenia and Autism: Implications for Cancer Comorbidity and Beyond

**DOI:** 10.3390/ijms17060856

**Published:** 2016-06-01

**Authors:** Enni Markkanen, Urs Meyer, Grigory L. Dianov

**Affiliations:** 1 Institute of Pharmacology and Toxicology, Vetsuisse Faculty, University of Zürich, Winterthurerstrasse 260, Zürich 8057, Switzerland; urs.meyer@vetpharm.uzh.ch; 2 Cancer Research UK and Medical Research Council Oxford Institute for Radiation Oncology, Department of Oncology, University of Oxford, Oxford OX3 7DQ, UK; grigory.dianov@oncology.ox.ac.uk; 3 Institute of Cytology and Genetics, Russian Academy of Sciences, Lavrentyeva 10, Novosibirsk 630090, Russia

**Keywords:** DNA base excision repair, XRCC1, neurodevelopmental disorders, oxidative DNA damage, oxidative stress, autism, schizophrenia, cancer

## Abstract

Schizophrenia and autism spectrum disorder (ASD) are multi-factorial and multi-symptomatic psychiatric disorders, each affecting 0.5%–1% of the population worldwide. Both are characterized by impairments in cognitive functions, emotions and behaviour, and they undermine basic human processes of perception and judgment. Despite decades of extensive research, the aetiologies of schizophrenia and ASD are still poorly understood and remain a significant challenge to clinicians and scientists alike. Adding to this unsatisfactory situation, patients with schizophrenia or ASD often develop a variety of peripheral and systemic disturbances, one prominent example of which is cancer, which shows a direct (but sometimes inverse) comorbidity in people affected with schizophrenia and ASD. Cancer is a disease characterized by uncontrolled proliferation of cells, the molecular origin of which derives from mutations of a cell’s DNA sequence. To counteract such mutations and repair damaged DNA, cells are equipped with intricate DNA repair pathways. Oxidative stress, oxidative DNA damage, and deficient repair of oxidative DNA lesions repair have been proposed to contribute to the development of schizophrenia and ASD. In this article, we summarize the current evidence of cancer comorbidity in these brain disorders and discuss the putative roles of oxidative stress, DNA damage and DNA repair in the aetiopathology of schizophrenia and ASD.

## 1. Schizophrenia and Autism Spectrum Disorder: Symptoms and Origins

Schizophrenia and autism spectrum disorder (ASD) are multi-factorial and multi-symptomatic psychiatric disorders, each affecting 0.5%–1% of the population worldwide [1,2]. Both are characterized by impairments in cognitive functions, emotions and behaviour, and they undermine basic human processes of perception and judgment [2,3]. In view of their chronicity and severity, schizophrenia and ASD impose tremendous mental and economic burdens on the affected individuals, and in turn, on their families and society in general [4,5].

Typically, the onset of full-blown schizophrenia is in early adulthood and includes a myriad of symptoms, which are commonly referred to as positive, negative and cognitive symptoms [2]. Positive symptoms are pathologies that do not present in healthy individuals but are a result of the disease process. These include visual and/or auditory hallucinations, delusions, paranoia, and psychomotor agitation. Negative symptoms refer to physiological features that are reduced or absent as a result of the disease process, including social withdrawal, apathy, deficits in motivation and reward-related functions, and alogia. Cognitive symptoms of schizophrenia involve deficits in executive functions, working memory, and attention, which in turn can undermine reasoning, planning and problem solving in affected individuals.

ASD is characterized, to a varying degree, by severe deficiencies in social interaction, a lack of verbal and nonverbal communication, and the presence of restricted and repetitive behaviours [3]. Initially, ASD was sub-categorized into autism, Asperger syndrome, pervasive developmental disorder-not otherwise specified (PDD-NOS), and childhood disintegrative disorder [1,3]. These subtypes seemed to differ primarily in the nature and/or severity of cognitive delay and the presence of intellectual disability. Individuals with Asperger syndrome generally lack delays in cognitive development and communication, in contrast to autism. With the release of the 5th Diagnostic and Statistical Manual of Mental Disorders (DSM-5), however, all subtypes were merged into one umbrella diagnosis of ASD [6]. Overt symptoms of ASD can begin as early as by the age of six months, become established by two to three years, and tend to persist throughout life [1,3,7,8,9,10].

Interestingly, schizophrenia and ASD seem to overlap at multiple levels. For example, disruption of emotional processing and sensorimotor gating, and impairments in executive functions, are psychopathological features commonly observed in both disorders [3,11,12,13,14,15,16]. Furthermore, patients with schizophrenia and ASD display a similar pattern of deficient neuronal activation during a social cognition task, with both patient groups showing reduced activation in the right amygdala, fusiform gyrus, and ventrolateral prefrontal cortex [17,18,19,20,21,22]. The impression of overlapping brain abnormalities between schizophrenia and ASD has also been supported by meta-analytic anatomical likelihood estimations consolidating multiple imaging datasets, which reveal appreciable brain structural concordance between the two disorders, especially in the right parahippocampal gyrus, posterior cingulate, putamen, claustrum and left thalamus [13,17,21,23]. Finally, the neuropathology of schizophrenia and ASD also overlap at the cellular levels. One example relates to the imbalances in excitatory *versus* inhibitory neurotransmission, which involve disturbances in glutamate and γ-aminobutyric acid (GABA) signalling and leads to increased excitatory-inhibitory ratios in both disorders [7,8,10,19,20,21].

Despite decades of extensive research, the aetiologies of schizophrenia and ASD are still poorly understood and remain a significant challenge to clinicians and scientists alike. According to the prevailing view, both disorders involve changes in early brain development and subsequent brain maturation [11,12,14,15,16,24]. Hence, an interaction between foetal neurodevelopmental disturbances and changes in postnatal brain maturation seems necessary to trigger the onset of full-blown psychotic or autistic disorders. Within this neurodevelopmental framework, the combined action of multiple genes (of small effect size) and a number of environmental risk factors is required to cause schizophrenia and ASD [17,19,20,21,22,25]. According to such gene-environment interaction models, the effect of an individual’s genotype depends on environmental exposure and, vice versa, the effect of environmental exposure on risk depends on an individual’s genotype [17,21,23,26,27]. Recent advances in genome-wide association studies (GWAS) have generated reproducible findings on a number of common risk alleles in both schizophrenia and ASD [19,20,21,28,29]. However, the heritability estimates of the relative contribution of common genetic variants based on molecular genetic data appear to be considerably smaller [24,30] than heritability estimates from twin studies [25,31,32]. The discrepancy between heritability estimates from twin and molecular genetic studies may emerge because gene-environment interactions involving shared environmental factors within families are included in heritability estimates of twin studies, but not in molecular genetic studies of unrelated subjects [26,27,33]. The comparison between monozygotic and dizygotic twins allows, to a certain extent, an estimation of the relative contribution of genetic *versus* environmental factors, and consequently, twin studies can readily take into account gene-environment interactions in their heritability estimates. Indeed, it is the general consensus that differences between monozygotic twins are primarily attributable to the environment, whereas differences between dizygotic twins to both hereditary and environmental factors in twin studies [34]. According to several lines of evidence, however, deducing the contribution of genetic factors in twin studies may be misleading, partly because the prenatal environment (and its interaction with genetic predisposing factors) is rarely dealt with adequately [35,36,37]. For example, the majority of monozygotic twins are monochorionic. Hence, many monozygotic twins share the same placenta, whereas all dizygotic twins are dichorionic [38]. Given that normal placental functions are essential for proper foetal development, including brain development [39], neurodevelopmental abnormalities arising from placental dysfunctions in monochorionic twins may be misinterpreted as being of genetic origin.

The further elucidation of aetiological mechanisms in schizophrenia and ASD also appears crucial with regards to the need of more effective treatments. In schizophrenia, pharmacotherapy with currently available antipsychotic drugs can only partially normalize psychopathological symptoms and are particularly poor in alleviate negative and cognitive symptoms [28,29,40]. Similarly, there are still no effective treatments against ASD-related symptoms such as social interaction and communication deficits [30,41].

Adding to this unsatisfactory situation, patients with schizophrenia or ASD often develop a variety of peripheral and systemic disturbances, some of which are clinically highly relevant and can further undermine the daily life quality of the affected individuals [31,32,42,43]. One prominent example is cancer, which shows a direct (but sometimes inverse) comorbidity in people affected with neurodevelopmental disorders such as schizophrenia and ASD [33,44,45,46,47]. In this article, we summarize the current evidence of cancer comorbidity in these brain disorders and discuss the putative role of DNA damage in this context.

## 2. Schizophrenia, Autism Spectrum Disorder and Cancer Risk

The hypothesis of altered cancer risk in schizophrenia is not new, yet it still appears conflicting, as reviewed in [40,41]. For example, early studies by Mortensen [42,43,48] showed that the overall incidence of cancer was lower for male (but not female) patients with schizophrenia as compared to the general population. A similar reduction in overall cancer risk has also been demonstrated in several subsequent studies (e.g., [44,45,46,47,49,50]. Interestingly, Ji *et al.* [47,51]) reported that the overall cancer risk was not only lower in patients, but also significantly reduced among their unaffected parents and siblings, suggesting the involvement of genetic factors in this association. A similar finding was provided by Catts *et al.* [47,52], who found reduced cancer risk in unaffected first-degree relatives of patient with schizophrenia.

These findings suggesting reduced cancer risk in schizophrenia contrast with other epidemiological reports demonstrating no changes or even increased risk of developing cancer in schizophrenia. For example, some reports show increased overall cancer risk in patients with schizophrenia [48,53,54] or a higher risk for specific cancers such as colon, breast and stomach cancers [49,50,55,56]. Increased cancer risk was also found in the study by Lin *et al.*, who demonstrated that this association is restricted to female patients with schizophrenia and inversely correlate with the age of schizophrenia onset [51,52]. Marked sex differences were also obtained in the study by Ji *et al.*, suggesting that female schizophrenic patients have a higher cancer risk than the general population for breast, cervical and endometrial cancers [47,48].

Hence, the extent to which schizophrenia is associated with altered cancer risk remains controversial (e.g., reviewed in [33,53,54,57]). One reason for these conflicting findings may be the varying contribution of environmental factors such as smoking habits, nulliparity, obesity and exposure to antipsychotics [55,56,58]. Indeed, adjustments for possible confounding factors such as smoking can markedly change the magnitude or even the direction of cancer risk. For example, Catts *et al.* found that schizophrenia is associated with a modest but significant increase in lung cancer risk in unadjusted analyses [52,59,60,61,62]. When adjusted for smoking, however, patients with schizophrenia tend to show decreased lung cancer risk. Similarly, Lichtermann *et al.* found that patients with schizophrenia display an increased overall cancer risk, whereas cancer risk is consistently lower in their unaffected first-degree relatives. These diametrically opposite effects may readily be explained by the varying contribution of specific lifestyle factors such as excessive smoking and alcohol consumption, which are more prevalent in patients with schizophrenia as compared to non-affected individuals [33,48,57].

In contrast to schizophrenia, only a few studies have estimated cancer risk in ASD. The available data suggest, however, that ASD is generally associated with increased cancer risk. For example, ovarian cancers and central nervous system (CNS) cancers such as neurofibromatosis are more prevalent in ASD subjects as compared to non-ASD subjects [33,57,63]. There is also initial evidence suggesting that ASD is associated with increased breast cancer risk [58,64]. Interestingly, recent genome-wide exome sequencing has revealed extensive overlap in risk genes for ASD and for cancer [59,60,61,62,63], suggesting that altered cancer risk in ASD subjects may have a strong genetic basis. Many of these genes have been implicated in pathophysiological processes underlying cancer development, including genes involved in DNA repair [33,57,65]. DNA repair is considered one of the foremost cellular defence mechanisms to counteract cancer formation, raising the possibility that changes in DNA repair and/or DNA damage might be a molecular link between altered cancer risk and neurodevelopmental disorders such as ASD and schizophrenia.

## 3. DNA Damage, DNA Repair and Cancer

Cancer is a disease characterized by the loss of control by the organism over a small subset of cells, which start to proliferate regardless of the borders imposed by the surrounding tissue. For this to occur, cancer cells have to acquire a subset of characteristics that ensures their survival and enables their growth despite adversary reactions from the surrounding. These features are collectively referred to as ‘hallmarks of cancer’ [63,66].

The molecular origin of virtually all cancers derives from mutations of a cell’s DNA sequence [64,67,68]. Indeed, genomic instability and mutation is recognized as one of the very few enabling characteristics that clearly drive cancer formation by facilitating the acquisition of all other core hallmarks of cancer [63,65]. Mutations can arise when the integrity of DNA is challenged by agents deriving from exogenous sources [65]. To counteract the formation of mutations, cells have evolved a plethora of DNA repair pathways that sense, report and correct alterations in DNA [66,69,70]. Deficiencies in some of these repair pathways have been implicated to act as key players in the induction and progression of cancer [63,64,67,68]. It is noteworthy, however, that even in the absence of exposure to damaging exogenous agents (such as ultraviolet (UV) radiation or tobacco smoke), DNA is a very sensitive molecule prone to undergo spontaneous alterations. These can be caused by DNA’s chemical instability as well as many different intracellular (endogenous) mutagens that are present in the cell (reviewed in [65,71]). For instance, the need for oxygen to support all vital functions of a mammalian cell drives also the formation of a variety of reactive oxygen products, such as hydrogen peroxide, superoxide ions or hydroxyl radicals. These reactive oxygen species avidly react with macromolecules present in the cell, among which DNA presents a particularly vulnerable target. DNA is prone to undergo spontaneous alterations, caused by its chemical instability, as well as the presence of endogenous harmful agents, such as oxygen radicals (reviewed in [65,68,72,73,74,75,76]). Such insults lead to the formation of small base lesions and single-strand DNA breaks (SSBs), which are constantly undermining genomic integrity. Estimates of these lesions range from 10,000–20,000 for small base lesions up to 150,000 for SSBs generated in every single cell every single day under physiological, non-stressed conditions [69,70,71]. If left unrepaired, this type of damage can give rise to the formation of mutations in the DNA, which can ultimately lead to the onset of diseases such as cancer [63,64,71,76,77].

There are multiple DNA different repair pathways that catalyse the reconstitution of DNA integrity when it has been compromised. Among them, base excision repair (BER) is one of the most important mechanisms to safeguard cells from the frequently occurring small DNA base lesions and SSBs [71,73]. BER acts like a housekeeper that constantly cleans away the multitude of different base lesions and SSBs, thus ensuring the maintenance of intact and unaltered double-stranded DNA, which in turn is pivotal for all physiological cellular functions. Taking into account the numbers of DNA lesions that BER has to correct even under physiological conditions, it follows that a decrease in BER repair capacity—either by changes in the protein sequence due to genetic polymorphisms, or changes in protein expression levels—can lead to an accumulation of DNA damage in the cells. Therefore, disruptions in DNA repair pathways can cause a change in DNA repair capacity of the affected cell or tissue that might manifest itself in increased levels of DNA damage. This readily predisposes cells to accumulating DNA damage, which in turn has been aetiologically linked to the development of a variety of human diseases, such as cancer, premature aging, and also neurodegenerative conditions like Alzheimer’s disease and Parkinson’s disease [68,71,72,73,74,75,76].

**Figure 1:**
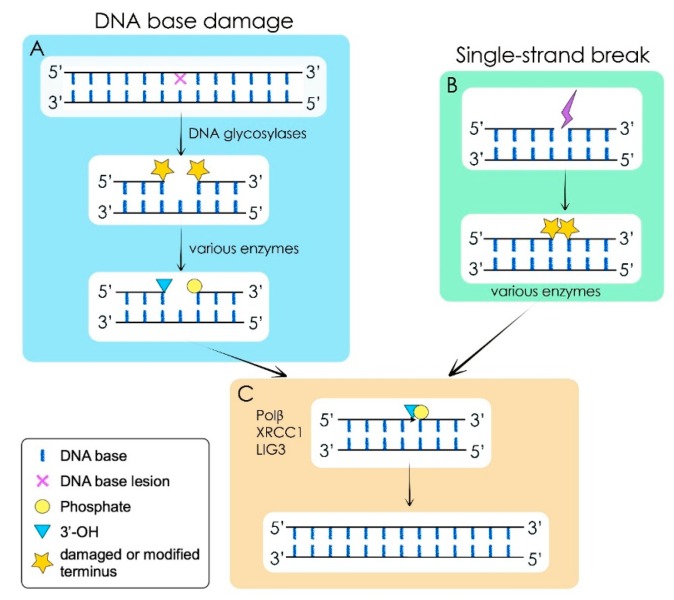
Simplified scheme of base excision repair (BER). Modified after [76,78]. (**A**) BER is initiated by damage-specific DNA glycosylases, which identify and release the corrupted base by hydrolysis of the *N*-glycosylic bond linking the DNA base to the sugar phosphate backbone (reviewed in [76,79]). The arising abasic (AP) site is further processed by AP-endonuclease 1 (APE1), and depending on the mechanism by which the DNA base was removed, end processing of the modified 3′- and 5′-termini is performed by a variety of end-processing enzymes. This processing results in the generation of a 3′-OH and a 5′-P group adjacent to the DNA gap or break; (**B**) Single-strand breaks (SSBs) can also arise from direct disintegration of oxidised deoxyribose. This process usually leads to damaged or modified termini, which are processed by a variety of enzymes to 3′-OH and 5′-P groups. SSBs are then handled identically to the BER intermediates from this point onward; (**C**) Further processing of the SSB containing intermediate stemming from either source is carried out by the core BER complex that includes DNA polymerase β (Pol β), XRCC1 (X-ray repair cross-complementation group 1) and DNA ligase IIIa (Lig III). Pol β possesses a dRP-lyase activity that removes the 5′-sugar phosphate and also, functioning as a DNA polymerase, adds one nucleotide to the 3′-end of the arising single-nucleotide gap. Finally, the XRCC1-Lig III complex seals the DNA ends, therefore accomplishing complete DNA repair [71,80,81,82]*.*

Classical BER of damaged DNA bases is initiated by damage-specific DNA glycosylases, which identify and excise the corrupted base by hydrolysis of the *N*-glycosylic bond linking the DNA base to the sugar phosphate backbone (reviewed in [71,76], for details, see Figure 1). The phosphodiester bond 5′ to the resulting abasic site (AP-site) is cleaved by AP endonuclease 1 (APE1), thus generating a single strand DNA break with 5′-deoxyribose phosphate. Importantly, different classes of DNA glycosylases exist, and they differ in their mechanism to release the damaged base, resulting in a variety of intermediate products with chemically different termini (for details, see [71,76,77]). All these repair intermediates containing a variety of termini have to be further processed to yield 3′-OH and 5′-P residues flanking the 1-nucleotide gap, as these are prerequisite to allow DNA polymerisation and ligation of the ends. There are several end-processing enzymes in mammalian cells including APE1, polynucleotide kinase phosphatase (PNKP) and aprataxin, which removes blocking residues from 5′- to 3′-DNA ends (reviewed in [71,73]). Further processing of this single strand break containing intermediate is carried out by a DNA repair complex that includes DNA polymerase β (Pol β), X-ray repair cross-complementation group 1 (XRCC1) and DNA ligase IIIa (Lig III). Pol β has a dRP-lyase activity that removes the 5′-sugar phosphate and also, functioning as a DNA polymerase, adds one nucleotide to the 3′-end of the single-nucleotide gap. Finally, the XRCC1-Lig III complex seals the DNA ends, therefore accomplishing DNA repair [71,73].

The enzymes of the BER pathway also repair DNA SSBs. SSBs arise from disintegration of oxidised deoxyribose, yielding a break in the DNA backbone while retaining all DNA bases. Very often, the termini resulting from such breaks are damaged, and thus need to be processed to give rise to 3′-OH and 5′-P DNA ends. This processing is performed by a variety of different enzymes, and the interested reader is referred to an excellent review on this subject [73,83]. After DNA end processing has been accomplished, the DNA ends are ligated back together by the Polβ/XRCC1/Lig3 complex.

Several mutations in the BER-pathway have been associated with the development of cancer (reviewed in [78,84]). Most notably, defects in repair of one of the most abundant oxidative DNA lesion, 8-oxo-deoxyGuanine (8-oxo-G), are implicated in familial cancer syndromes involving mutations in the DNA glycosylases MutY homolog (MUTYH) or 8-oxoguanine DNA-glycosylase 1 (Ogg1). 8-oxo-G is a miscoding lesion that can lead to insertion of incorrect A opposite 8-oxo-G upon DNA replication, which therefore frequently leads to formation of an A:8-oxo-G base pair during DNA replication (reviewed in [79,85]). If left unrepaired, this mismatch can further propagate into a CG→AT transversion point mutation upon further replication. MUTYH is a DNA glycosylase that removes the A from A:8-oxo-G base pairs, thus paving the way for correction of the 8-oxo-G damage by a coordinated interplay of DNA polymerase λ, Ogg1 DNA glycosylase and the canonical BER pathway [80,81,82,86,87,88].

As mentioned above, cancer is a disease characterized by uncontrolled proliferation of cells. In view of the fact that ASD and schizophrenia are manifestations of neuronal cell dysfunction, how can a defect in a single gene possibly cause neuronal dysfunction—which *per se* is not characterized with increased cellular proliferation, as neuronal cells are postmitotic—while at the same time causing an defect in another tissue that gives rise to cancer? In an attempt to answer this, consider the following examples.

Several genetic disorders demonstrate that changes in a single protein can have a multifaceted impact on the entire organism. Among the most prominent examples to illustrate that a defect in one gene product gives rise simultaneously to a variety of neurological as well as oncological disorders is Ataxia-Teleangiectasia (A-T). A-T is a rare autosomal recessive disorder caused by mutation of the gene A-T mutated (ATM), a serine-threonine protein kinase that orchestrates the response to cellular stress situations, such as DNA double-strand breaks (reviewed in [83]). A-T patients develop early-onset progressive cerebellar ataxia, oculocutaneous teleangiectasia, immunodeficiency, and have a high incidence of cancer, particularly lymphoid tumours. One of the main questions arising from these findings is, how can a gene defect cause cellular dysfunction in one tissue, while being accessory to the development of a proliferative malignancy in others? The main reason for this variegated phenotype is most likely based on cellular differences in the affected tissues, namely postmitotic neurons in the brain *versus* proliferative cells from the hematopoietic lineage in the case of lymphoid tumours. While genetic instability in postmitotic cells such as the brain manifest in cellular dysfunction, which then leads to clinical features of neurodegeneration due to the loss of neuronal tissue, in a cell with replicative potential it can lead to malignant transformation thus giving rise to cancer. Another very interesting example for how a decrease in neuronal function can be associated at the same time with an increased cancer risk are patients with Down syndrome (DS). In DS patients chromosome 21, or parts thereof, is present in triplicate, which clinically manifests in physical growth delays, intellectual disability, and a series of other disorders (reviewed in [84]). Moreover, DS patients exhibit a 10- to 20-fold higher risk for leukemia but markedly lower incidence of solid tumours (reviewed in [85]). The molecular basis behind these differences in cancer predisposition remain unclear to date, but the cell-type specific effect that is alluded to above could very well play an important determinant in the case of DS as well.

## 4. Oxidative Stress, DNA Damage and DNA Repair in Schizophrenia and Autism Spectrum Disorder

### 4.1. Oxidative Stress, Mitochondrial Dysfunctions and DNA Damage

As outlined above, DNA damage can either stem from exposure to exogenous DNA damaging agents, such as tobacco smoke or UV radiation, endogenous sources such as oxidative stress stemming from the respiratory chain, or it can be caused by a decrease in repair of normal levels of DNA damage constantly present in our genomes. Within the last decades, a plethora of findings have implicated oxidative stress and subsequent DNA damage in the development of schizophrenia and ASD. Indeed, signs of increased oxidative stress and oxidative DNA damage has been detected in various tissues of schizophrenic patients [86,87,88,89,90,91,92,93,94,95,96,97,98,99,100,101,102,103,104,105,106,107]. Additional evidence supporting a role of redox imbalances and oxidative stress in the development of schizophrenia stems from various animal models, which suggest that increased oxidative stress during sensitive windows of brain development and maturation is causally related to the subsequent emergence of schizophrenia-related brain and behavioral abnormalities [108,109,110,111,112].

Redox imbalances and oxidative stress in schizophrenia may further involve, or even be triggered by, functional deficits in the mitochondrial respiratory chain (reviewed in [108,113]). In support of this hypothesis, mitochondrial dysfunctions have been closely linked to the pathogenesis of schizophrenia and related psychotic disorders (reviewed in [89,90,114,115]). It still remains debatable, however, whether increased oxidative stress is the primary cause or rather a consequence of mitochondrial deficits. According to recent theories [91,93,95,116], oxidative stress may not be primarily involved in generating mitochondrial DNA damage, but rather triggers a cellular stress response that in turn causes damage. It is also known that deletions in mitochondrial DNA increase as a function of age, especially in post-mitotic cells with high metabolic demands, such as the brain. Interestingly, however, post-mortem analyses of mitochondrial deletions revealed a significant decrease in the global accumulation of mitochondrial DNA deletions in patients with schizophrenia as compared to patients with major depressive disorder or bipolar, or compared to age-matched control subjects [108,117]. This decrease was largest in dopaminergic regions such as caudate nucleus, putamen and substantia nigra [108,118]. One interpretation of these findings is that schizophrenia is, for yet unknown reasons, associated with a primary deficit in mitochondrial functions, which in turn might lead to a decrease in mitochondrial metabolism and therefore result in reduced mitochondrial deletions.

Increased levels of oxidative stress and oxidative DNA damage have also been reported in ASD subjects, as well as in animal models relevant to this disorder [114,115,119]. Reduced ratios of reduced glutathione (GSH) to its oxidised form GSSG (glutathione disulfide), indicative of oxidative stress, along with decreased total GSH levels were found in the cerebellum and temporal cortex in ASD subjects, suggesting a deficit in the redox balancing system and the antioxidant defence mechanisms in affected individuals [116,120]. Decreased GSH:GSSG ratios along with increased levels of 8-oxo-G and other markers of oxidative stress were also detected in the cerebellum and Brodmann Area 22 of ASD subjects [117,121,122,123]. Moreover, oxidation damage to DNA and a deficit in antioxidant capacity was also found in plasma from children with ASD as compared to non-affected siblings [114,118,124].

Similar to schizophrenia, functional deficits in the mitochondrial respiratory chain may readily contribute to the emergence of increased oxidative stress in ASD. In support of this hypothesis, Napoli *et al.* found higher frequencies of deletions and small base transitions in the mitochondrial DNA obtained from ASD children [119,125]. Signs of deficient mitochondrial functions in ASD subjects are also apparent in post-mortem brain samples. For example, Tang *et al.* revealed altered levels of proteins involved in the mitochondrial respiratory chain, decreased activity of the respiratory chain, decreased antioxidant levels, as well as more oxidative DNA damage in post-mortem brains of patients affected with ASD [120,126].

### 4.2. Dysregulated DNA Repair and DNA Damage

Insufficiency in DNA repair is an alternative (but not mutually exclusive) mechanism that can precipitate DNA damage in general, and in neurodevelopmental disorders such as schizophrenia and autism in particular. Indeed, several studies have linked polymorphisms in DNA repair genes to the development of schizophrenia and ASD. Intriguingly, genetic polymorphisms in the core BER-protein XRCC1 itself, or in XRCC1 interacting proteins such as Ogg1, have been repeatedly implicated in schizophrenia [121,122,123,127] and ASD [114,124,128]. The mRNA levels of the DNA glycosylase MBD4 and Ape1 were also reported to be upregulated in the brains of patients with schizophrenia and related disorders [42,43,51,125], adding further evidence for the hypothesis of altered DNA repair in psychotic disorders.

In an effort to assess the cellular capacity for DNA repair in schizophrenia patients, several attempts have been made to measure DNA damage under basal conditions and in response to cellular stressors. Quantification of DNA repair through measurements of unscheduled DNA synthesis in response to methyl methanesulfonate (MMS), methylnitronitrosoguanine (MNNG) and UV radiation [126,129], or through the Comet assay after H_2_O_2_ treatment or γ-irradiation [127,130], failed to detect any significant differences between cells obtained from patients with schizophrenia and controls. Young *et al.* reported similar results, yet they found higher levels of DNA damage in male compared to female patients [128,131]. The latter results emphasize the importance of assessing phenotypes in a gender-specific manner, similarly to what has been proposed for epidemiologic investigations assessing cancer incidence in schizophrenia (e.g., [42,43,51,132]). Flow cytometric analysis of the DNA double-strand break marker γH2AX in immortalized lymphoblasts from schizophrenia patients revealed a significantly higher baseline levels of γH2AX in untreated cells, and a reduced γH2AX response upon irradiation with 5 Gray [63,64,67,68,129]. Higher baseline levels of γH2AX in untreated cells may mirror pre-existing DNA damage as a result of increased oxidative stress and/or replication stress, whereas a failure to mount sufficient γH2AX responses may point towards an aberration of the DNA damage response involving γH2AX formation in lymphoblasts from schizophrenic patients. It should be noted, however, that γH2AX is only a surrogate marker for cellular stress responses but does not index DNA damage *per se.* Hence, the extent to which these findings may reflect actual differences in DNA repair still awaits verification.

Attempts to explore DNA repair mechanisms in ASD are limited to a few investigations and have thus far provided equivocal results. For example, cytogenetic analyses of chromosome fragile sites initially revealed significant differences in chromosome lesions in ASD patients relative to controls [130,133]. In contrast, a comparison between lymphocytes from ASD children, unaffected siblings and community controls showed no signs for genomic instability using the cytokinesis-block micronucleus assay (CBMA) [76,131]. While the latter assay allows detection of gross molecular events like chromosome breakage, loss or rearrangement, it is unable to detect more subtle changes in DNA structure and repair. Therefore, these findings leave the question unanswered as to whether ASD patients may display more subtle damage to DNA or not. Comparing lymphoblasts from ASD children and their siblings, Main *et al.* further found an increased sensitivity of ASD samples to necrosis caused by oxidative stress. At the same time, the authors revealed no difference in DNA damage using the CBMA assay [132,133], suggesting that ASD patients do not display gross abnormalities in DNA structure and repair.

Taken together, there is accumulating evidence supporting a role of increased oxidative stress and impaired mitochondrial functions in schizophrenia and ASD. However, the available data are inconclusive, so that additional research will be needed to ascertain whether or not DNA repair mechanisms are altered in these disorders. Whilst genetic linkage studies readily support this hypothesis, the nature and/or severity of abnormal DNA repair and damage in schizophrenia and ASD await further exploration.

## 5. DNA Repair and Damage in Schizophrenia and Autism: A Role beyond Altered Cancer Risk?

In view of the crucial roles of genomic stability and DNA repair in cancer [63,64,67,68,133], it appears plausible that altered expression of DNA repair genes and/or functional impairments in DNA repair mechanisms may contribute to altered cancer risk in schizophrenia and autism. Exploring this putative relationship clearly warrants future investigation as it may help advance our understanding of why certain types of cancers are more prevalent in patients with schizophrenia or ASD, whereas others seem to be less common in these neurodevelopmental disorders (see Section 2). Another burning question in this context is whether altered DNA repair and/or DNA damage may play an aetiopathological role beyond cancer risk. Or in other words: is there a pathological connection between altered DNA repair pathways and abnormal brain development relevant to the neurodevelopmental origins of schizophrenia and ASD?

Experimental work in animal models has recently begun examining this question. Bjørge *et al.* used a mouse model, in which the expression of the two DNA glycosylases Ogg1 and MUTYH was genetically ablated [133,134]. As discussed above, Ogg1 and MUTYH are both involved in the removal of the oxidative DNA lesion 8-oxo-G, so that their ablation are expected to increase DNA damage in dividing cells [76]. The authors found that loss of both these glycosylases induced a synergistic effect on anxiety-like behaviour [133,135,136,137]. Transcriptomic analysis of the hippocampi of these mice revealed a possible role of the two glycosylases in hippocampal gene expression, which related primarily to mal-adaptive behaviour. However, Ogg1 and MUTYH double-knockout animals did not display overt changes in 8-oxo-G levels in any of the brain tissue analysed [133,138], making it difficult to attribute the observed behavioural and transcriptional changes to DNA damage. An alternative (and more likely) explanation for the behavioural induced by Ogg1 and MUTYH ablation is that the genetic manipulation interfered with transcription independently of 8-oxo-G processing in the brain. Furthermore, the lack of 8-oxo-G accumulation following Ogg1 and MUTYH could be the result of redundancies in pathways catalysing its removal, an effect that may be particularly pronounced in the CNS due to the postmitotic nature of brain cells.

Selected deficits in learning and memory have also been observed in mice deficient of other DNA glycosylases, including Neil1 and Neil3 [134,139] and reviewed in [76,140]. Even though the relative contribution of altered DNA repair and DNA damage remains elusive in these models, these findings indicate that the loss of these DNA glycosylases exerts a long-lasting impact on cognitive functions. Based on these promising findings, extending the phenotypes of Neil1 and Neil3 ablation to other behavioural and cognitive functions relevant to schizophrenia and ASD, including social interaction, central information processing and filtering, sustained and selective attention, and working memory, seems highly warranted [135,136,137,141].

Long-lasting neural defects have also been identified in mice with genetic XRCC1 deficiency [138]. A reduction of XRCC1 levels leads to a decrease in the overall BER capacity of a cell, primarily because Pol β and Lig III are unstable in the absence of XRCC1 as scaffolding protein [139,142,143]. This leads to the accumulation of endogenous SSBs in primary cells, even in the absence of exogenously administered DNA damaging agents, as endogenously arising lesions are not repaired properly and in time [140]. The complete knockout of XRCC1 is embryonically lethal, possibly because of a failure to repair DNA breaks arising during embryonic development [141]. To circumvent this problem, Lee *et al.* (2009) developed an XRCC1-loxP mouse that allows a conditional ablation of XRCC1 in specific tissues when crossed with the appropriate Cre-driver mouse expressing the cre-recombinase in the tissue of interest [138]. Using this approach, they showed that Nes-Cre driven ablation of XRCC1 in the CNS leads to impaired development of the cerebellum and hippocampus, two brain regions strongly implicated in neurodevelopmental disorders such as schizophrenia and ASD [142,143]. These abnormalities seemed to arise from a persistent accumulation of single-strand strand breaks in mature neuronal populations and markedly affect γ-aminobutyric acid (GABA) interneurons [138]. The latter effects are of particular interest because cellular abnormalities in GABAergic circuitries are hallmark neuropathological features of neurodevelopmental brain disorders such as schizophrenia and autism [144,145]. GABAergic interneurons critically regulate neuronal oscillatory activity [146], which in turn is believed to serve various complex functions, including perception, cognition, and memory [147]. Hence, impairments in GABAergic functions resulting from genetic XRCC1 deficiency may cause a spectrum of behavioural and cognitive deficits that are reminiscent of functional deteriorations in developmental neuropsychiatric disorders. Whether DNA damage is directly involved in the aetiopathology of schizophrenia and ASD, however, still awaits direct examination.

## 6. Conclusions and Perspectives

To summarize, direct experimental evidence for the hypothesis that DNA damage and/or genetic disruptions of DNA repair genes can causally contribute to the onset of schizophrenia and ASD is slowly growing. Nevertheless, whether DNA damage and DNA repair is causally connected to ASD and schizophrenia remains controversial. Indeed, the current status of research is still far from rendering even a rudimentary picture of all the possible mechanisms involved, or understanding how, and in which phase, which particular source of DNA damage leads to alterations relevant to schizophrenia and autism. Here, it will be highly interesting and important also to dissect the contribution of the DNA damaging insult occurring during prenatal development *versus* damage incurred throughout adolescence or adulthood. Furthermore, it remains completely unclear how DNA damage mechanistically drives cellular changes that drive the observed phenotype in these disorders. Another salient feature in respect to patient-based epidemiological studies is the observation of gender-specific differences pertaining to certain traits, as seen for cancer incidence reports. Future research—not only in this field, but also in other biomedical research—will probably have to address these gender-specific variations more thoroughly when assessing phenotypes, both in epidemiological as well as in basic research settings. Detailed analysis of all these aspects warrants future investigations to broaden our understanding of the underlying mechanisms, and has the potential to spark development of novel therapeutic approaches to cure, or at least alleviate the severity, of neurodevelopmental diseases.
